# Effects of Forest Type on Nutrient Fluxes in Throughfall, Stemflow, and Litter Leachate within Acid-Polluted Locations in Southwest China

**DOI:** 10.3390/ijerph19052810

**Published:** 2022-02-28

**Authors:** Zhan Chen, Yihao Wang, Ruisi Chen, Xiuya Ni, Jixin Cao

**Affiliations:** 1 Key Laboratory of Forest Ecology and Environment of National Forestry and Grassland Administration, Institute of Forest Ecology, Environment and Nature Conservation, Chinese Academy of Forestry, Beijing 100091, China; nxya0517@caf.ac.cn (X.N.); caojx@caf.ac.cn (J.C.); 2 College of Geography and Tourism, Chongqing Normal University, Chongqing 401331, China; 20190053@cqnu.edu.cn; 3 School of Mathematics and Physics, University of Science and Technology, Beijing 100083, China; 42021045@xs.ustb.edu.cn; 4 Beijing Academy of Forestry and Landscape Architecture, Beijing 100044, China

**Keywords:** throughfall, stemflow, litter leachate, soil acidification, *Cinnamomum camphora*, *Pinus massoniana*

## Abstract

Although new inputs of acidic anions are decreasing, soil acidification still deserves more academic attention because of the effects of historical stores of SO_4_^2^^−^ already absorbed into soils. Forest canopy has large, species-specific effects on rainwater chemistry, for which the hydrological mechanism remains unclear. We investigated precipitation, throughfall, stemflow, and litter leachate across three forest types in a severely acid-polluted site located in Southwest China. Precipitation monitored over 4 months, representing summer, fall, winter, and spring, indicated neutral precipitation in Tieshanping with pH ranging from 6.58–7.33. Throughfall and litter leachate in *Pinus massoniana* Lamb. stands were enriched with greater cation and anion fluxes, as well as more dissolved organic carbon (DOC) flux. Rainwater from pure stands of *Cinnamomum camphora* (Linn) Presl yielded lower N and DOC inputs to soils with higher base saturation, which would reduce soil acidification and, therefore, improve the sustainability of forest ecosystems.

## 1. Introduction

Acid deposition is a global environmental problem that has garnered great interest due to its negative impacts on ecosystems [1,2]. Since the early 1980s, the government has made efforts to decrease acidic emissions, especially SO_2_ linked to acid deposition, yet nitrogen oxide emissions have been increasing with the rapid increase in the use of private cars for nearly two decades, so it still remains a severe issue in southern China [2,3,4]. Soil acidification caused by acid deposition has adverse effects on ecosystems, particularly forest ecosystems [5]. Numerous studies report the damage of acid deposition on forests, including decreased growth and dieback [6], changing biogeochemical cycles [7], altered aboveground and belowground biomes [8,9,10], and impacts on ecosystem function and sustainability [11,12]. Additionally, despite reductions of acidic anion inputs [13,14], the recovery of acidified soils could be delayed due to historic adsorption of SO_4_^2−^ into soils [1]. Soil properties, including soil acidity, are highly affected by forest types [15,16].

Actually, the process of the effects of forest canopies on soil nutrients is very complex. On the one hand, forest covers can alter microclimatic conditions [17], including the surface albedo, temperature, wind and humidity, which may affect decomposition rates [18], soil properties, and further affect plant growth as a feedback loop [17]. The effects of forests on the surface albedo and temperature are also complicated and result in various effects in different latitudes with tropical, temperate or boreal forests [19,20]. Additionally, forest canopies change precipitation partitioning that affects the nutrient cycling in the forest ecosystem: interception, throughfall, and stemflow [21]. Canopy partitioning can impact the dynamics of tree water and nutrient balances [22]. The minerals dissolved in interception can directly enter the plant through foliar and bark uptake, and also can reach forest soil via throughfall or stemflow. Nutrients can also be leached from throughfall and stemflow and input into soils [22]. Interception as well as throughfall and stemflow are influenced by many features of the forest type, such as density, branch angle, the uniformity, bark characteristics, leaf shape and leaf area index [23], which are very complicated in forest ecosystems. Further, the chemical enrichment of throughfall and stemflow and nutrient leaching capacity exhibit considerable species-specific variability [24], which further complicates general conclusions on the effects of forest types on water and soil properties, especially under the background of climate change, because forests interact with climate change via albedo, evaporation, the carbon cycle and other factors [25,26].

Inputs of acidic anions and other nutrients to the forest floor through precipitation are affected by dry deposition on the canopy and the canopy itself, both of which can alter water chemistry and further affect soil quality. Throughfall and stemflow supply available nutrients to the forest floor, which can affect soil acidity [27], although the volume of stemflow is much less than that of throughfall and often ignored [28]. Before nutrient-carrying throughfall and stemflow enter into the soil, they pass through the layer of forest floor litter (except in areas of bare ground), where nutrients from throughfall, stemflow, and litter combine to form litter leachate, which ultimately affects soil quality. A study in British Columbia confirmed that throughfall and stemflow contribute to higher pH, N availability, and total exchangeable bases in forest floor areas associated with bigleaf maple, which have legacy effects on soil fertility [29]. Forest floor leachate chemistry largely determines biogeochemical processes in the soil and soil solution chemistry, affecting soil acidity and the sustainability of the forest ecosystem [30]. It is necessary and important to account for forest floor leachate in addition to throughfall and stemflow when studying the hydrological effects of rainfall on soil in forested ecosystems.

The exchange capacity of basic cations and acid anions in forest canopy and litter have strong species specificity [24,27,31,32]. Individual tree species play an important role in the distribution and cycling of nutrients that influence ecosystem function, biodiversity, resistance to stress, and sustainability [33,34]. Previous studies have reported that different forest types can strongly affect the chemical composition of throughfall, stemflow, and floor leachate, particularly the effect of nitrate and sulfate on anionic charges [24,27,31,32,35]. It was reported that the canopy of conifers intercepted particles and cloud droplets more efficiently than deciduous species, resulting in generally higher dry deposition under conifers [36,37]. Deciduous forests receive less S and N than coniferous stands via throughfall plus stemflow on the forest floor as reviewed by De Schrijver et al. [31]. This is attributable to the higher dry deposition capacity of conifers, which were also indicated to be more vulnerable to acidification with higher seepage of NO_3_^−^, SO_4_^2−^, and cations compared to deciduous stands [38,39]. Previous studies revealed higher DOC and H^+^ fluxes and lower pH and exchangeable cations in forest floor leachate from conifers compared to broadleaved stands [27,30,40]. Thus, while throughfall, stemflow, and forest floor leachates may play an important role in influencing acidity and nutrients of soils, their importance has been rarely studied [32].

Tieshanping, located in the southwest of China, is one of the most severely acid-polluted sites in China. Stands of *Pinus massoniana* (Pi) that naturally regenerated between 1958 and 1962 showed decreased growth attributed to acid deposition. A native deciduous species, *Cinnamomum camphora* (Linn) Presl (Ci), was planted between the 1980s and 1990s to improve forest health. A pure plantation of Ci was established with mixed stands of Pi and Ci (Pi_Ci) and pure Pi stands to test the impacts of different tree species on soil acidification. Numerous studies were performed in this hot spot, analyzing N deposition, forest health, soil quality, and litter decomposition [1,6,41,42]; however, the forest hydrology studies conducted in this location focused only on throughfall in Pi stands [1,43,44,45]. Therefore, this canonically acidic site provides an opportunity to study the effects of precipitation, throughfall, stemflow, and floor leachate on soil acidity in different forest types and fill the knowledge gap on this topic.

In this study, our objectives were to ascertain the impacts of different waterflow types and forest canopy types on nutrient dynamics in southwest China. We hypothesized that (1) compared to decades ago, the pH of precipitation in Tieshanping has increased and transformed from sulfate acid rain to nitrate acid rain; (2) the influence of throughfall on the nutrient cycle is greater than that of stemflow; (3) a plantation of broadleaved species (Ci) is best to improve the quality of acidic soil, followed by mixed deciduous and conifer, and pure conifer stands.

## 2. Materials and Methods

### 2.1. Study Area and Plot Design

The experiment was conducted in Tieshanping Forest Park (106°41.24′ E, 29°37.42′ N), which was a heavily polluted “acid rain control zone” located about 25 km northwest of Chongqing in subtropical southwest China. In June 2018, in each of the three different forest stands (Pi, Pi_Ci, Ci) we established four 20 m × 20 m square study plots. The details about the study site can be found in Chen et al. [10]. The soil is classified as Haplic Acrisol in FAO (IUSS Working Group WRB, 2006), with low pH values (3.80–4.54 from the O horizon to the lower A horizon) [10].

### 2.2. Collection and Analysis of Water Samples

**Figure 1:**
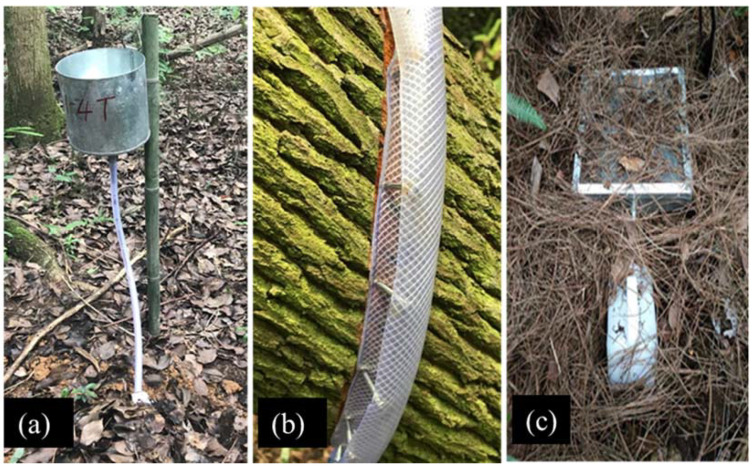
Water sample collectors: (**a**) throughfall, (**b**) stemflow, (**c**) litter leachate.

Bulk precipitation, throughfall, stemflow, and litter leachate were sampled as per Wang et al. [46] and measured in July 2018, October 2018, January 2019 and April 2019, which represent summer, autumn, winter, and spring, respectively. Bulk precipitation and throughfall were collected using stainless steel funnels (20 cm diameter) placed about 1.5 m above the forest floor. Three collectors were placed in an open field outside of the forest perimeter to measure bulk precipitation. For throughfall measurement (Figure 1a), five collectors were randomly placed in each plot. The funnels drained into opaque 10 L plastic buckets, which were placed below the ground surface. The stemflow (Figure 1b) was collected by 2 cm o.d. Tygon tubing with a length of about 100 cm. Before installing the tubing, the bark was shaved to ensure the seal without damage to the cambium layer. The tubing was split longitudinally and wrapped spirally around the tree stem, fastened with staples, sealed to the bole with acrylic caulk, and inserted into a 10 L plastic bucket. Litter leachate (Figure 1c) was collected using a topless 30 cm × 30 cm × 8 cm (length × width × height) stainless steel cuboid, which was covered with wire netting (1.5 mm mesh size). Each collector was connected with tubing to a 10 L plastic bucket, which was placed below ground level. The collectors were installed directly under the litter layer with five replicates per plot.

All water samples were collected weekly in the four sampling months and lumped into monthly samples for chemical analysis (stored at 4 °C before pooling). In total, there were 12 samples for precipitation and 16 samples, respectively, for throughfall, stemflow, and litter leachate for each forest type spanning July 2018 to April 2019.

All samples were filtered through 0.45 μm member filters before analyses. Water pH and electrical conductivity (EC) were measured by a benchtop pH meter (REX PHS-3C, Shanghai, China) and a benchtop conductivity meter (REX DDS-12A, Shanghai, China), respectively. We used a total organic carbon analyzer (TOC-L, SHIMADZU, Kyoto, Japan) to determine the dissolved organic carbon in all samples. The content of Ca and Mg cations was analyzed by an atomic absorption spectrophotometer (AAS, HITACHI ZA-3300, Tokyo, Japan), and K and Na concentration was measured using a flame photometer (FP640, Shanghai, China). An ion chromatograph (ICP, Dionex ICS-900, USA) was used to determine the anion concentration in all samples. NO_3_-N and NH_4_-N were measured using a flow analyzer (Seal AA3, Germany).

### 2.3. Data Curation and Statistical Analysis

Ion concentration was represented as ratio of cations to anions (RCA), calculated as ∑^+^ (sum of K^+^, Na^+^, Ca^2+^, Mg^2+^, NH_4_^+^ concentration) to ∑^−^ (sum of F^−^, Cl^−^, SO_4_^2−^, NO_3_^−^ concentration). The ion fluxes were calculated by multiplying ion concentrations by water fluxes. Base cation and anion fluxes (∑^+^ flux and ∑^−^ flux) were, respectively, the sum of K^+^, Na^+^, Ca^2+^, Mg^2+^, NH_4_^+^, and F^−^, Cl^−^, SO_4_^2−^, NO_3_^−^.

Water pH, EC, DOC, and RCA were analyzed by using one-way ANOVA with Tukey’s HSD to determine the different ion concentration among forest types respectively for stemflow, throughfall, and litter leachate. For each forest type, one-way ANOVA, Tukey’s HSD was also used to detect the seasonal variation across water properties. Independent *t*-tests were performed to determine significant differences across water properties, between precipitation and stemflow, throughfall, or litter leachate. All figures were created in OriginPro 9.0.

## 3. Results

### 3.1. Precipitation

The pH values of precipitation varied from 6.58 to 7.33 in the four sampling months, which indicated the precipitation was not acid rain in these months. The pH, EC and DOC contents had similar seasonal variation with highest values in January (Table 1).

**Figure 2:**
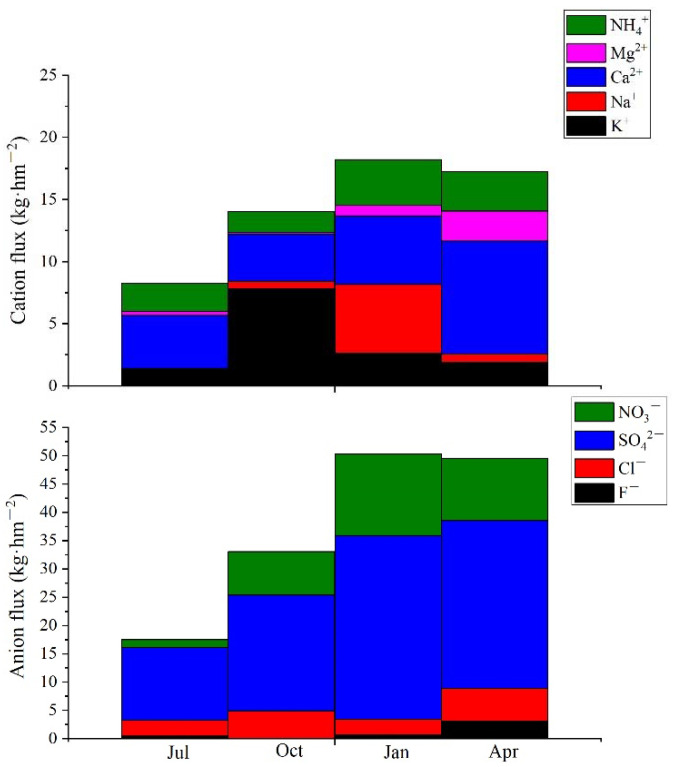
Cation and anion flux in precipitation.

Ca^2+^ was proportionally the most abundant cation, ranging between 24.81–71.62%, and SO_4_^2−^ was the most abundant anion (varying from 55.65–66.05%), followed by NO_3_^−^ (11.02–22.73%). There was no significant seasonal variation for RCA in precipitation (*p* = 0.862) (Table 1). Following the peak ion concentration in January, cation and anion flux also peaked in January (Figure 2). Ca^2+^ flux predominated in July and April (respectively 51.16% and 52.53% of the whole cation flux), while K flux was greatest in October with 7.84 kg·hm^−2^. In January, Ca and Mg flux combined accounted for about 30% of the whole cation flux. SO_4_^2−^ flux accounted for 59.94–73.34% of all anion flux (Figure 2).

### 3.2. Throughfall, Stemflow, and Litter Leachate

#### 3.2.1. pH, EC, and DOC

**Figure 3:**
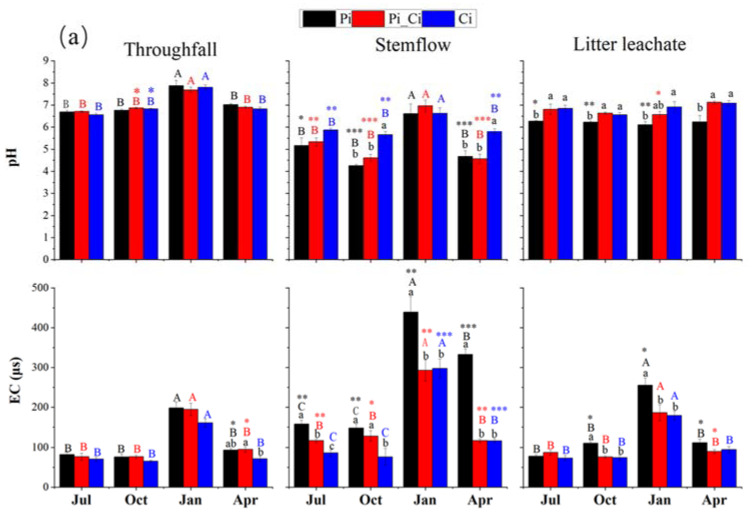

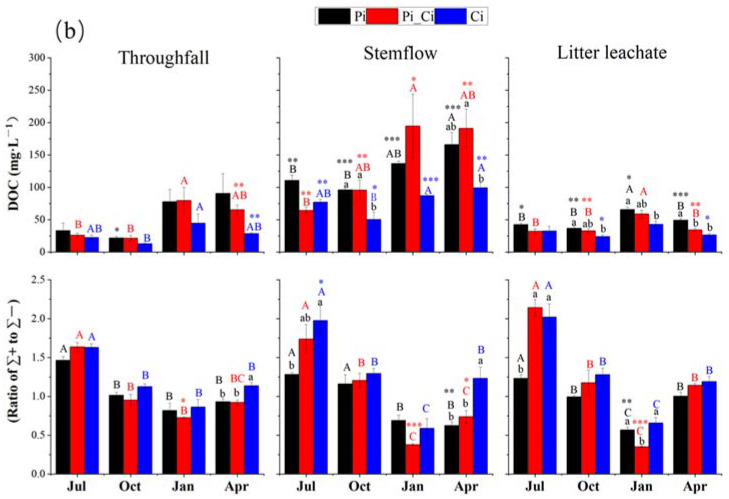
pH, electrical conductivity (**a**), dissolved organic carbon, and ratio of ∑^+^ to ∑^−^ (RCA) (**b**) of throughfall, stemflow, and litter leachate in different forest types during the sampling months. Capital letters indicate significant differences between sampling times for waterflows of each forest type. Lowercase letters indicate significant effects of forest type at the same sampling time. Asterisks indicate significant differences between precipitation and stemflow, throughfall, and litter leachate at the same sampling time. *, ** and *** respectively indicate significance at *p* < 0.05, 0.01 and 0.001.

The pH values (6.56–7.87) of all throughfall samples were slightly higher than that of precipitation, although not statistically significant (*p* > 0.05), with the exception of the October samples in Pi_Ci and Ci. The pH values in all stemflow samples were significantly lower than that of precipitation (*p* < 0.05), with the exception of the January samples. Forest types had significant effects on stemflow pH (*p* < 0.001), but not on throughfall pH (*p* = 0.491). Stemflow pH was about 5.6 in Pi and Pi_Ci, except for the January samples, while the pH of stemflow in Ci varied from 5.66–6.62. There was significant seasonal variation in pH values for stemflow (*p* < 0.001) and throughfall (*p* < 0.001), with significantly higher values in January. There were no observed seasonal effects on the pH of litter leachate (*p* = 0.061), whereas forest type had a significant effect (*p* < 0.001). The pH values of litter leachate in the Pi_Ci and Ci plots were significantly (*p* < 0.05) higher than that in the Pi plot, except for Pi_Ci sampling in January (Figure 3a).

Both EC and DOC of stemflow were significantly greater compared to those of precipitation (*p* < 0.05), except for EC of Ci in July and October. Forest type had opposite effects on EC and DOC concentration compared to pH of stemflow and litter leachate, with unclear impacts on throughfall. In Pi stands, the EC of stemflow (ranging from 147.7 to 439.25 μs) was significantly higher than that in Pi_Ci (117.50–292.88 μs) and Ci (76.45–297.75 μs) during all the sampling months (*p* < 0.05), except for the October samples in Pi_Ci. Among all water samples, the DOC concentration in precipitation was the lowest, with an average of 24 mg·L^−1^, compared to higher DOC levels in throughfall, litter leachate, and especially stemflow. Forest type did not impact DOC of throughfall. DOC concentration in stemflow (mean of 114.18 mg·L^−1^) was significantly higher than in precipitation in all plantations from all sampling months (*p* < 0.05). DOC concentration in stemflow from Ci (50.28–99.41 mg·L^−1^) was lower than that from Pi_Ci (64.44–194.57 mg·L^−1^) and Pi (96.34–166.16), and the difference was statistically significant in October (*p* = 0.027) and April (*p* = 0.032). Similarly, DOC concentration in Ci litter leachate was significantly lower than that in the Pi_Ci and Pi (*p* < 0.001) litter leachate samples (Figure 3b).

DOC flux in throughfall for Pi and Pi_Ci was triple and double that of precipitation in April, respectively. Due to lower volume, DOC flux in stemflow was reduced compared to that in precipitation, despite the higher concentration of DOC in stemflow. DOC flux in litter leachate was lower than that in precipitation, except in July, and was much lower than that in throughfall. Throughfall DOC in Ci was lower by 46.70%, 34.27%, 42.70% and 55.59% in July, October, January, and April, respectively, and litter leachate DOC flux was significantly lower, by 41.58%, compared to Pi in April (*p* = 0.027) (Table 2).

#### 3.2.2. Ion Concentration and Flux

**Figure 4:**
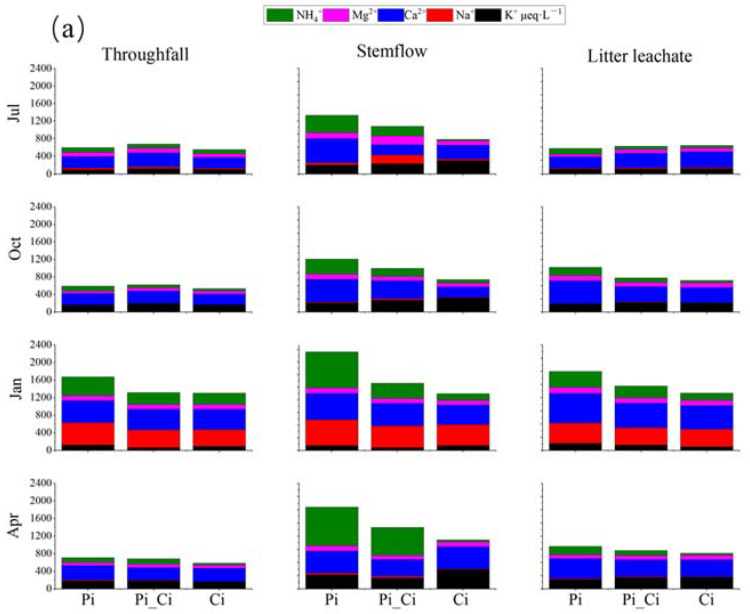

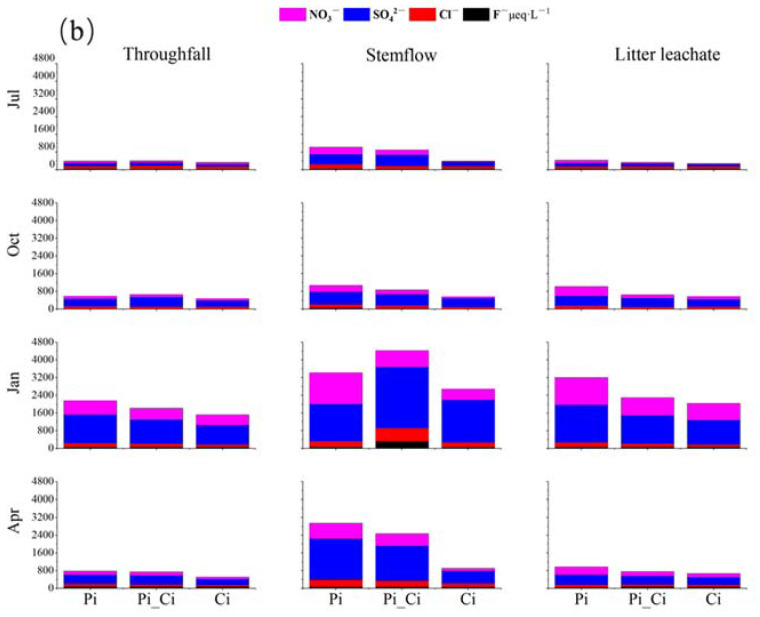
Cation (**a**) and anion (**b**) concentrations in throughfall, stemflow, and litter leachate among different forest types during different sampling months.

Most cation and anion contents were higher in concentration in throughfall, litter leachate, and especially stemflow, compared to precipitation. Ca^2+^ and SO_4_^2−^ were, respectively, the most abundant cation and anion (Figure 4). The mean sum of cation concentration was 605, 535.53, 966.26 and 662.97 μeq·L^−1^, and the mean sum of anion concentration was 755.94, 872.13, 1812.54 and 1110.88 μeq·L^−1^ in precipitation, throughfall, stemflow, and litter leachate, respectively (Figure 4).

We also found that the mean sum of cation or anion concentration could be ranked in the order Pi > Pi_Ci > Ci. In Ci, the greater difference in anions (lower by 32.87% and 39.08% compared to Pi_Ci and Pi) than cations (lower by 15.37% and 27.28% than Pi_Ci and Pi) resulted in higher RCA in Ci (Figure 3b). RCA in July was significantly higher than that in the other sampling months for all forest types (*p* < 0.05), except for stemflow sampling in October in Pi. Generally, RCA in Ci was larger than that in Pi, and the difference was statistically significant in July for stemflow (*p* = 0.025) and litter leachate (*p* = 0.011), and in April for stemflow (*p* = 0.005) and throughfall (*p* = 0.014). The mean value of RCA in Ci was greater by 35.60%, 12.52% and 35.53% compared to Pi, and by 25.26%, 12.29% and 6.97% compared to Pi_Ci for stemflow, throughfall, and litter leachate, respectively (Figure 3b).

**Figure 5:**
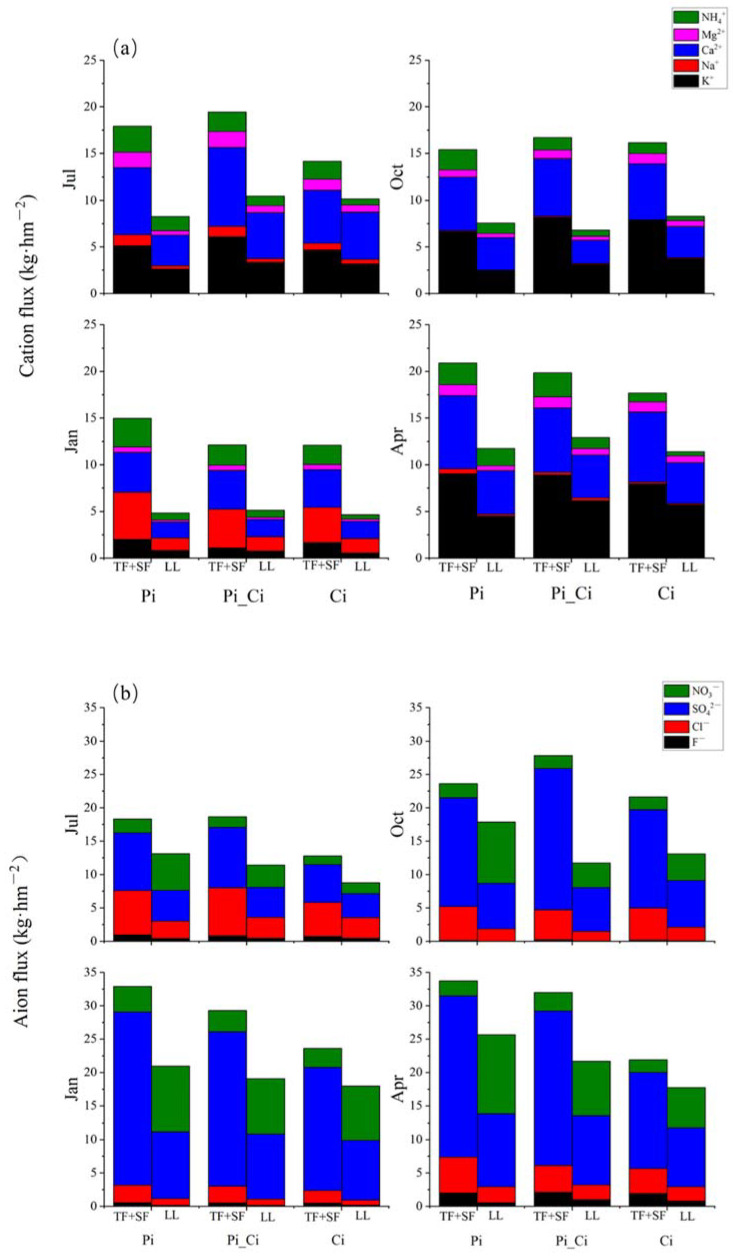
Cation (**a**) and anion (**b**) flux in throughfall plus stemflow and litter leachate. TF: throughfall; SF: stemflow; LL: litter leachate.

Due to the low nutrient flux (0.01875 to 0.3695 and 0.03950 to 0.6302 kg·hm^−2^, respectively, for cation and anion flux) in stemflow, in Figure 5 we show the flux as throughfall plus stemflow (TF+SF) and litter leachate (LL). The litter layer intercepted nutrient flux from forest rainwater, with the exception of NO_3_^−^. The leaf litter in Pi stands leached the most NO_3_^−^ (3.39–9.60 kg·hm^−2^), which was much greater than that from Ci stand litter (0.34 to 5.24 kg·hm^−2^). Due to different capacities for leaching NO_3_^−^ and intercepting SO_4_^2−^, litter leachate of Ci showed the lowest flux of NO_3_^−^ plus SO_4_^2−^ (5.23, 10.99, 17.04, and 14.79 kg·hm^−2^), followed by Pi_Ci (7.84, 10.24, 17.96 and 18.47 kg·hm^−2^), and then Pi, with the highest values observed (10.1, 15.98, 19.79, and 22.74 kg·hm^−2^) in July, October, January, and April, respectively.

The cation and anion fluxes of TF + SF in Ci were lower than those in Pi_Ci and Pi (Figure 5), except in October, while the ratio of cation flux to anion flux of throughfall and stemflow in Ci was significantly higher than that in Pi in July and April (Table 3). Compared to Ci stands, the ratio of cation flux to anion flux in litter leachate of Pi_Ci was greater by 17.39–46.03%. In Ci stands, the ratio of cation flux to anion flux was 80.95%, 39.53% and 36.17% greater than that in Pi stands in July, October, and April, respectively (Table 3). Additionally, for stemflow and litter leachate, base saturation in Ci was significantly higher than that in Pi, except for litter leachate in January, and also significantly higher than that in Pi_Ci, except for stemflow in January and litter leachate in July and January (Table 4).

## 4. Discussion

### 4.1. The Change of Precipitation

The data in this study indicated that precipitation in Tieshanping was neutral with pH values varying from 6.58–7.33. To evaluate the change in precipitation quality, we compared our data with previous data from 2001–2004 [47] and 2003 [48] in the same study site (Table 1). The mean pH values from 2001–2004 [47] and 2003 [48] were about 4.1, which was more acidic than the mean pH observed in this study, from 2018–2019, by over 2 units, and this could be due to decreased acidic emissions in China [1]. Most of the average ion concentrations substantially increased compared to mean values from 2001–2004, especially for K^+^ (3.5×), Ca^2+^ (8.6×), Cl^−^ (6.8×), SO_4_^2−^ and NO_3_^−^ (6–7×). In 2003 [48], most ion concentrations were much lower than those of 2001–2004, except for Ca^2+^, SO_4_^2−^ and NO_3_^−^, which were still lower than the values observed in this study. The ratio of SO_4_^2−^ to NO_3_^−^ in our study was an average of 3.16, which was much lower than that of the other two previous studies, at 4.27 and 5.3, respectively, in Xiang et al. [47] and Wang and Xu [48] in the same study site in Tieshanping. This increasing proportion of NO_3_^−^ in precipitation compared to previous studies [47,48] was also confirmed by Chen [41] in Chongqing. The increased RCA also confirmed the transformation of precipitation from acidic towards neutral. These data partially confirm our first hypothesis that the pH of precipitation and the proportion of NO_3_^−^ have increased. However, due to the large interannual variability observed over 2001–2004 [47], the 1-year monitoring data in this study are not sufficient to assert that precipitation in Tieshanping is presently neutral, not acidic. In order to do so, more observational data over a longer period would be needed.

### 4.2. The Effects of Water Flow

In our study, both cation and anion fluxes in litter leachate were lower than that in throughfall plus stemflow, which indicated an interception of nutrients at the litter layer. To further detect how much throughfall and stemflow influence the nutrient flux of litter leachate, we performed variation portioning analysis (VPA) using cation, anion, and DOC flux. As indicated by VPA, a total of 68% of litter leachate nutrient flux could be explained by throughfall and stemflow. Nutrient flux from throughfall and stemflow respectively contributed 20% and 3% to litter leachate nutrient flux, while their common contribution was 45%. The contribution of throughfall and stemflow to the forest floor has also been confirmed by previous data [29,32]. The relatively small contribution of stemflow observed in this study is supported by Bellot et al. [49], which found that nutrient inputs of stemflow only represented about 2–3% of total dissolved inputs into the soils. Despite the low contribution, stemflow can play an important role in forest ecosystems, as it concentrates water in the area immediately proximal to the tree trunk, providing a supply of water and nutrients to the tree and soil far more so than throughfall [49,50].

This present study also indicates that stemflow is enriched with a greater nutrient concentration than throughfall. We found the highest DOC content, cation and anion concentration, and lowest pH value in stemflow, followed by throughfall and litter leachate. Higher concentrations of nutrients in stemflow can be attributed to both nutrients leaching from stems and very low stemflow volume [51]. When we replaced nutrient flux with concentration in VPA analysis, the contribution of stemflow to litter leachates increased from 3% to 9%, which further illustrates the importance of stemflow in influencing nutrient concentrations in the root zone that affect rhizosphere microbial communities and function [52]. Thus, our second hypothesis is partially correct in that throughfall does contribute a greater nutrient flux to the forest floor than stemflow; however, the transient impacts of stemflow are more intensive on the root zone, specifically.

### 4.3. The Effects of Forest Type

A previous review that analyzed data from 17 stand pairs determined that coniferous forests intercept more pollutants than deciduous stands [31], a conclusion that our findings support as well. In this study, cation and anion flux in Ci were much lower than in Pi and Pi_Ci in throughfall plus stemflow; however, Ci had the highest pH and RCA and the lowest DOC in stemflow across three forest stands. A study in northwest China illustrated that the enrichment capacity of acid anions and exchangeable bases in the canopy of conifers is stronger than that of broadleaved species [27]. While these results were consistent with ours, Han et al. [27] recommended planting coniferous species to improve forest ecosystem function, a measure that may not be suitable in our study site. The study site in Han et al. [27] was located in northwest China, which does not experience acid rain or soil acidification [53]. Our study was conducted in a typical acid-polluted area with severely acidic soil (average pH of 3.98, 4.54, and 4.6, respectively, in O_horizon of Pi, Pi_Ci and Ci, [10]), which would benefit from species that could supply less acidic rain water and greater base saturation. We also calculated the base saturation of throughfall, stemflow, and litter leachate, finding that Ci had the highest base saturation, followed by Pi_Ci (Table 4). We also found that the sum of anion flux in litter leachate from Ci was much lower than that of Pi, which was mainly due to a lower amount of NO_3_^−^ flux in Ci. The lower flux of NO_3_^−^ from litter leachate in Ci than in Pi observed in this study is consistent with results from central Japan, which showed that average NO_3_^−^ flux of litter leachate in evergreen forests was higher than that in deciduous forests [54]. Our data showed that throughfall N input was negatively associated with soil pH, which is supported by a study from an urban-to-rural transect in southern China [55]. In this study, throughfall NO_3_^−^ flux was the highest in Pi stands, and the lowest in Ci stands, where lower N input of throughfall (plus stemflow) and litter leachate contributed to higher pH values of the soils, an observation supported by our previous data as well [10].

We also found that the DOC concentration and flux in throughfall and litter leachate were minimum in Ci and maximum in Pi. Though DOC flux in stemflow of Ci was relatively higher, it was negligible because of its small volume compared to throughfall and litter leachate. In our study, the DOC concentration in throughfall and DOC fluxes in forest floor leachates were found to be higher in coniferous species stands than in broadleaved species stands, which was supported by some previous studies [29,40,56]. DOC could influence water acidity due to the composition of short-chain acids and large molecules such as humic and fulvic acids [57]. Previous studies indicated that tree species with high DOC in their forest floor leachates stimulate base cation leaching, which would cause soil acidification [58].

In summary, Ci is more beneficial for improving soil acidification than Pi based on the acidic status of soils in Tieshanping. However, our findings are only based on measurements from four 1-month periods, which limited our knowledge of the effects of forest types on soil acidity. Longer-term and more-detailed monitoring data on different forest types in this study area will be needed in the future to further ascertain the effects of different water flow and forest types on soil quality.

## 5. Conclusions

Our monitoring data indicated that precipitation in Tieshanping was neutral and the proportion of NO_3_^−^ was increased. Despite very low nutrient fluxes, stemflow also played an important role in affecting the nutrient cycling in the area near the stem by increasing nutrient concentrations. According to hydrological chemical characteristics, pure plantations of Ci could supply lower N and DOC inputs to soils with higher base saturation for improving soil acidity, followed by mixed Ci and Pi stands. In stands of Ci, higher RCA and base saturation accompanied by lower N and DOC inputs in throughfall plus stemflow and litter leachate yielded higher pH in water flow entering the soils, which would support recovery from soil acidification and further ensure sustainable development of forest ecosystems in acid-polluted locations. This study underscores the changing rainwater chemistry of forest hydrological processes and the effects on soil acidification across mixed and pure stands of coniferous species and broadleaved species. While these findings are based on four 1-month periods of measurement, to clearly determine the water chemistry of precipitation and the effects of forest type in Tieshanping, more observational data over a longer period are needed.

## Figures and Tables

**Table 1:** pH, EC (μs·cm^−1^), DOC concentration (mg·L^−1^) and ion concentration (μeq·L^−^^1^) in precipitation.

	pH	EC	DOC	K^+^	Na^+^	Ca^2+^	Mg^2+^	NH_4_^+^	F^−^	Cl^−^	SO_4_^2−^	NO_3_^−^	SO_4_^2−^: NO_3_^−^	RCA
Jul-18	6.58B	65.87B	28.77AB	38.46	1.45B	248.15AB	29.43B	28.96B	32.26BC	105.23	231.60B	45.69B	2.81	1.00
Oct-18	6.58B	58.90B	10.76B	84.62	21.74B	59.01B	9.12B	63.38B	3.91C	95.9	297.33B	85.31B	3.54	0.81
Jan-19	7.33A	146.63A	41.01A	69.23	363.77A	416.00A	106.10A	292.60A	58.60AB	114.39	1018.62A	350.52A	2.87	0.85
Apr-19	6.77AB	66.40B	18.23B	28.21	18.84B	340.50A	109.03B	92.95B	83.50A	82.73	325.21B	92.95B	3.4	1.03
Mean	6.82	84.45	24.69	55.13	101.45	265.92	63.42	119.47	44.57	99.56	468.19	143.62	3.16	0.92
2001–2004 [47]	4.12			15.74	104.14	30.76	18.46	267.42		12.72	87.14	20.41	4.27	/
2003 [48]	4.1			8	3	58	9	76	5	11	184	35	5.3	0.6

**Table 2:** The flux of DOC in precipitation, throughfall, stemflow and litter leachate. BP: bulk precipitation; TF: throughfall; SF: stemflow; LL: litter leachate. Different lowercase letters indicate significant difference among forest types.

		DOC Flux (kg·hm^−2^)			
		Jul 2018	Oct 2018	Jan 2019	Apr 2019
	BP	11.99	15.45	27.52	34.65
TF	Pi	49.32	23.49	33.68	79.40 a
	Pi_Ci	34.06	23.53	35.90	77.63 a
	Ci	26.29	15.44	19.30	35.26 b
SF	Pi	0.4733	0.3038	0.0495	0.9595
	Pi_Ci	0.1863	0.2153	0.0828	0.7275
	Ci	0.7494	0.3070	0.1030	1.003
LL	Pi	27.42	12.33	8.29	25.78 a
	Pi_Ci	24.10	11.86	9.87	20.32 ab
	Ci	23.49	11.41	7.19	15.06 b

**Table 3:** Ratio of cation flux to anion flux across three forest types during four sampling months. Different lowercase letters indicate significant difference among forest types.

		∑^+^:∑^−^ (Flux)			
		Jul 2018	Oct 2018	Jan 2019	Apr 2019
TF	Pi	1.32 ± 0.038 b	0.65 ± 0.030	0.46 ± 0.065	0.63 ± 0.038 b
	Pi_Ci	1.32 ± 0.060 b	0.61 ± 0.048	0.41 ± 0.006	0.63 ± 0.027 b
	Ci	1.60 ± 0.099 a	0.75 ± 0.031	0.52 ± 0.021	0.81 ± 0.025 a
SF	Pi	1.16 ± 0.11 b	0.74 ± 0.094	0.45 ± 0.061 a	0.37 ± 0.025 b
	Pi_Ci	1.22 ± 0.082 b	0.98 ± 0.17	0.19 ± 0.010 b	0.49 ± 0.021 b
	Ci	2.05 ± 0.28 a	0.88 ± 0.048	0.33 ± 0.077 ab	0.85 ± 0.097 a
LL	Pi	0.63 ± 0.043 b	0.43 ± 0.012 b	0.23 ± 0.018	0.47 ± 0.025 b
	Pi_Ci	0.92 ± 0.13 ab	0.60 ± 0.059 a	0.27 ± 0.022	0.60 ± 0.018 a
	Ci	1.14 ± 0.11 a	0.63 ± 0.037 a	0.27 ± 0.032	0.64 ± 0.026 a

**Table 4:** Base saturation ((K^+^ + Ca^2+^ + Mg^2+^): ∑^+^) in throughfall, stemflow and litter leachate across different forest types. Different lowercase letters indicate significant difference among forest types.

	Jul 2018	Oct 2018	Jan 2019	Apr 2019
Throughfall				
Pi	75.58	80.79 b	44.85	83.12
Pi_Ci	81.21	87.99 a	48.85	81.98
Ci	77.80	89.96 a	51.39	91.49
Stemflow				
Pi	66.59 c	70.58 b	37.42 b	50.56 b
Pi_Ci	74.21 b	78.85 b	45.57 ab	58.08 b
Ci	92.17 a	90.09 a	51.55 a	93.75 a
Litter leachate				
Pi	72.86 c	81.27 b	54.43	78.66 b
Pi_Ci	85.03 ab	85.00 b	55.02	85.27 b
Ci	87.22 a	91.09 a	57.97	93.36 a

## Data Availability

The data presented in this study are available from the corresponding author upon reasonable request.
